# Supplementary material for: A closer look at four-dot masking of a foveated target
Source: PeerJ. 2016 Jun 2;4:e2068. doi: 10.7717/peerj.2068 (PMC4893326; doi:10.7717/peerj.2068)
Supplement: Supplemental Information 1 [file peerj-04-2068-s002.jasp › index.html]

JASP 


# Results

## Bayesian T-Test

| Bayesian Paired Samples T-Test | | | | | | | | | |
| --- | --- | --- | --- | --- | --- | --- | --- | --- | --- |
|  | |  | |  | | BF₁₀ | | error % | |
| Com Off |  | - |  | 250 ms |  | 8.617 |  | 1.121e -9 |  |
|  | | | | | | | | | |

### Inferential Plots

#### Com Off - 250 ms

##### Prior and Posterior

##### Bayes Factor Robustness Check
