# Supplementary figures and images for: A closer look at four-dot masking of a foveated target
Source: PeerJ. 2016 Jun 2;4:e2068. doi: 10.7717/peerj.2068 (PMC4893326; doi:10.7717/peerj.2068)

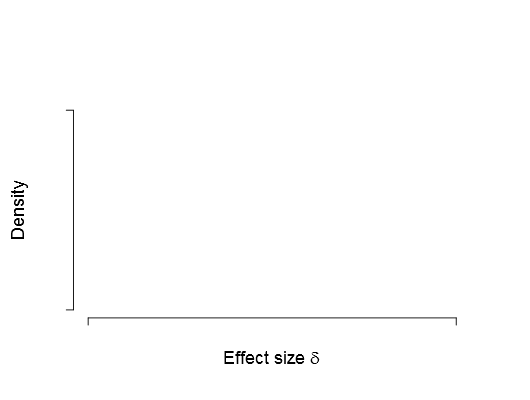

Supplement: Supplemental Information 1 [file peerj-04-2068-s002.jasp › resources/0/_5.png]

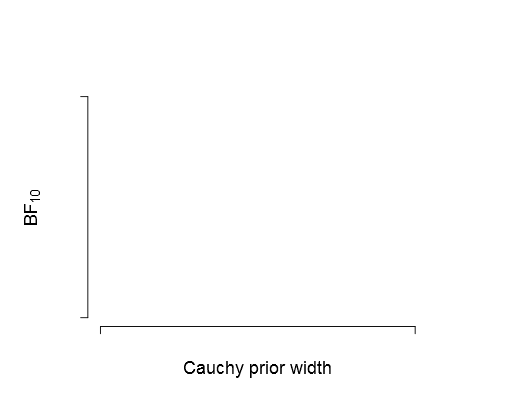

Supplement: Supplemental Information 1 [file peerj-04-2068-s002.jasp › resources/0/_6.png]

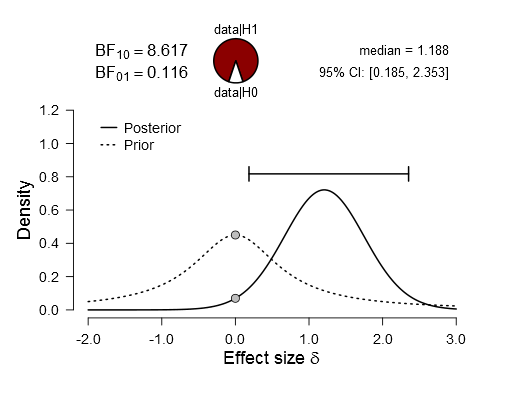

Supplement: Supplemental Information 1 [file peerj-04-2068-s002.jasp › resources/0/_7.png]

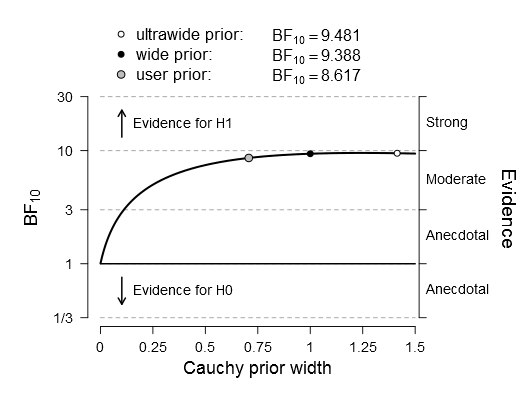

Supplement: Supplemental Information 1 [file peerj-04-2068-s002.jasp › resources/0/_8.png]

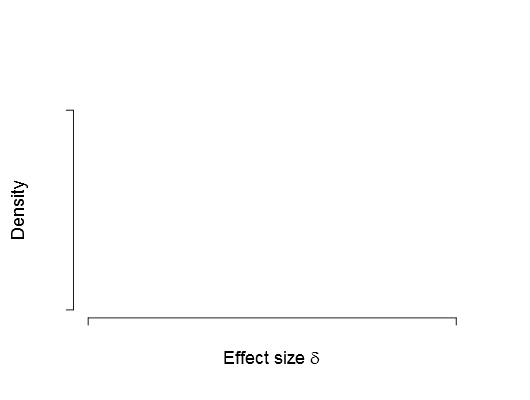

Supplement: Supplemental Information 3 [file peerj-04-2068-s004.jasp › resources/0/_5.png]

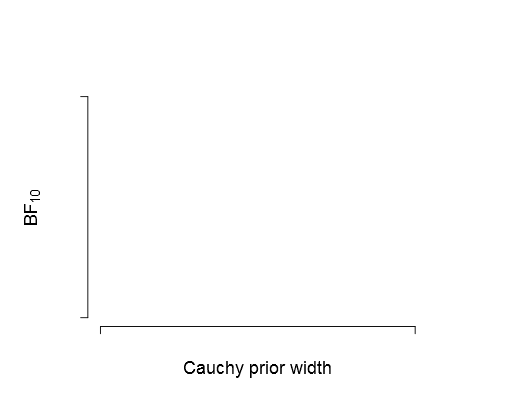

Supplement: Supplemental Information 3 [file peerj-04-2068-s004.jasp › resources/0/_6.png]

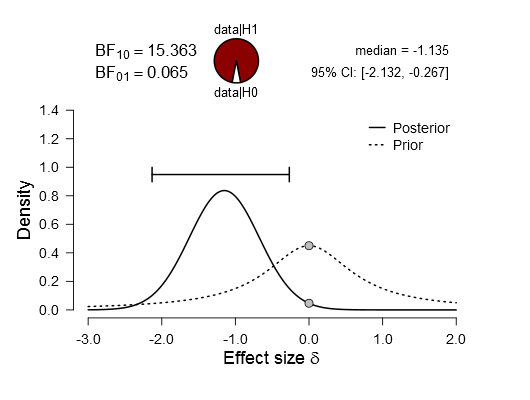

Supplement: Supplemental Information 3 [file peerj-04-2068-s004.jasp › resources/0/_7.png]

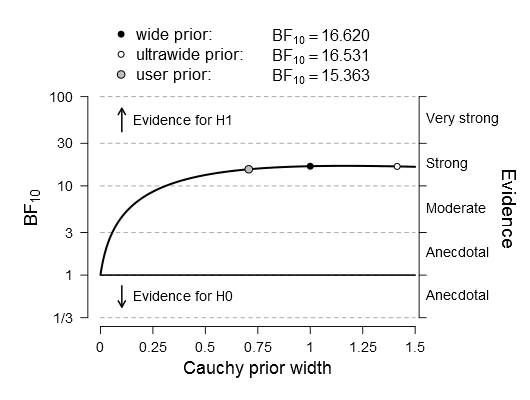

Supplement: Supplemental Information 3 [file peerj-04-2068-s004.jasp › resources/0/_8.png]

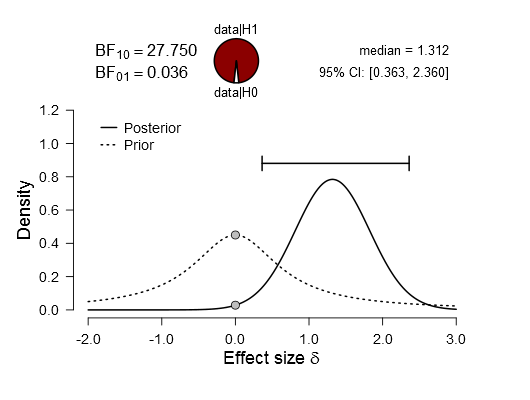

Supplement: Supplemental Information 4 [file peerj-04-2068-s005.jasp › resources/0/_19.png]

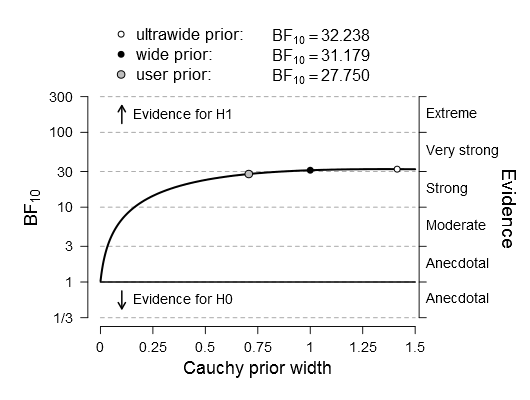

Supplement: Supplemental Information 4 [file peerj-04-2068-s005.jasp › resources/0/_20.png]

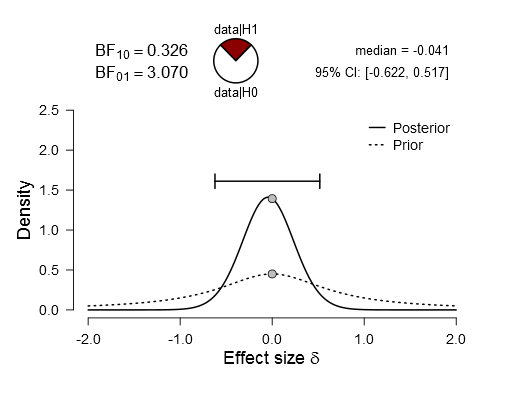

Supplement: Supplemental Information 4 [file peerj-04-2068-s005.jasp › resources/0/_21.png]

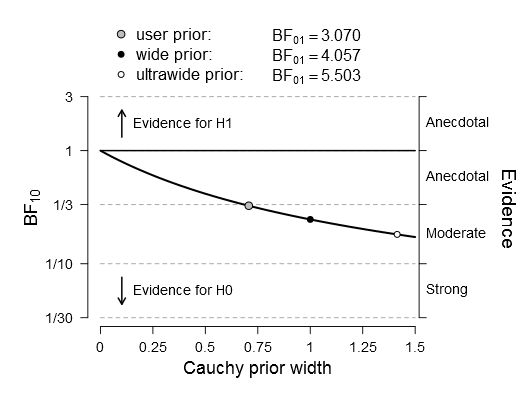

Supplement: Supplemental Information 4 [file peerj-04-2068-s005.jasp › resources/0/_22.png]

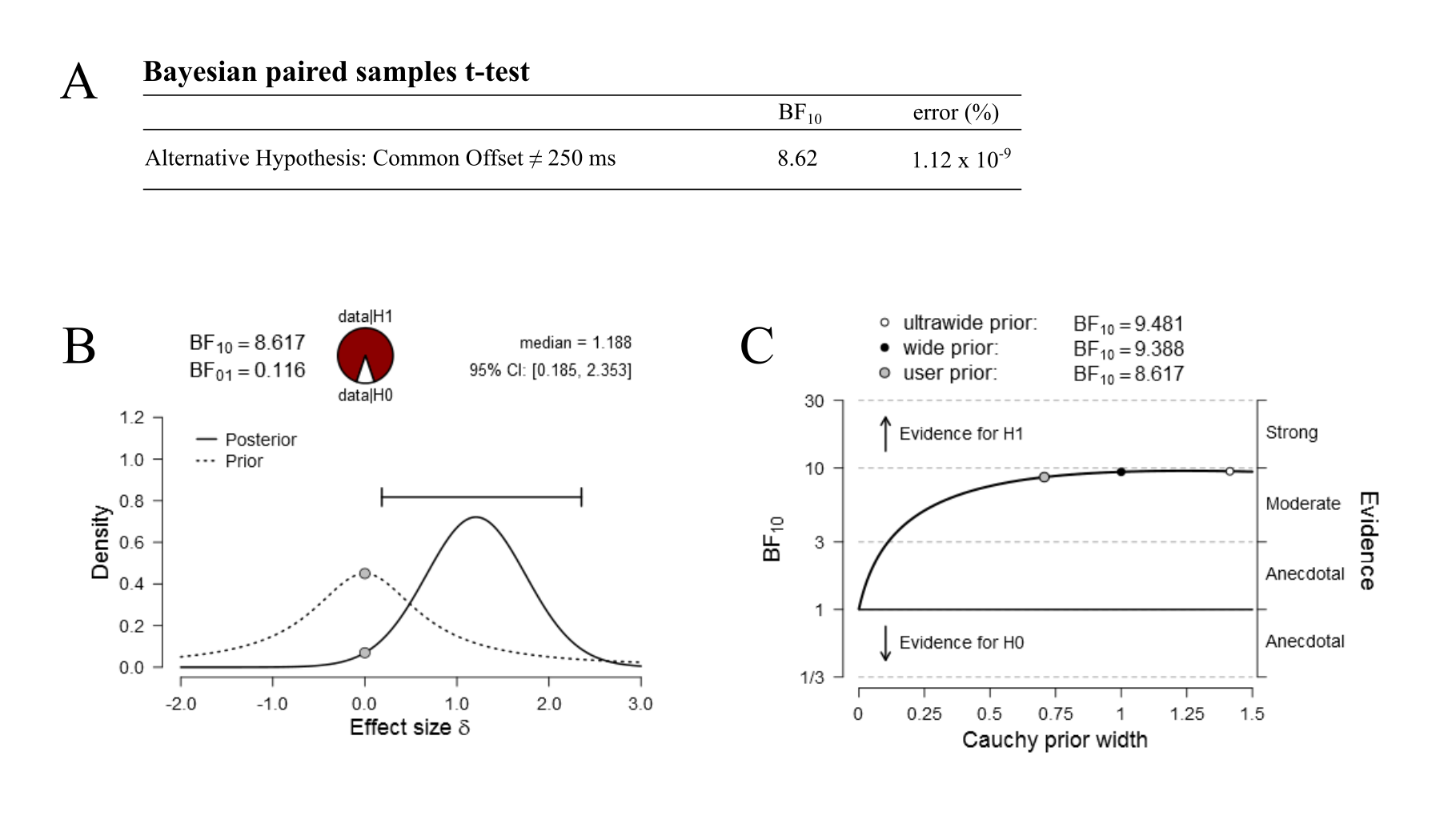

Supplement: Figure S1 — A: BF10 denotes the Bayes factor for the alternative hypothesis over the null. Here, the alternative hypothesis is that performance in baseline is different from that in the 250 ms condition. The null is that performance is equal in these two conditions. The error column indicates the error in estimating the Bayes factor, as a percentage. Shown in B are both the posterior and prior distributions of effect size. The null hypothesis defines an effect size of 0, depicted here in filled grey circles. C shows the robustness of the Bayes factor as a function of the prior width. The small grey circle depicts the Bayes factor at the default prior with of 0.707. The curve here shows that the Bayes factor remains relatively stable until much lower widths are used. B and C were generated using the JASP software. [file peerj-04-2068-s007.png]

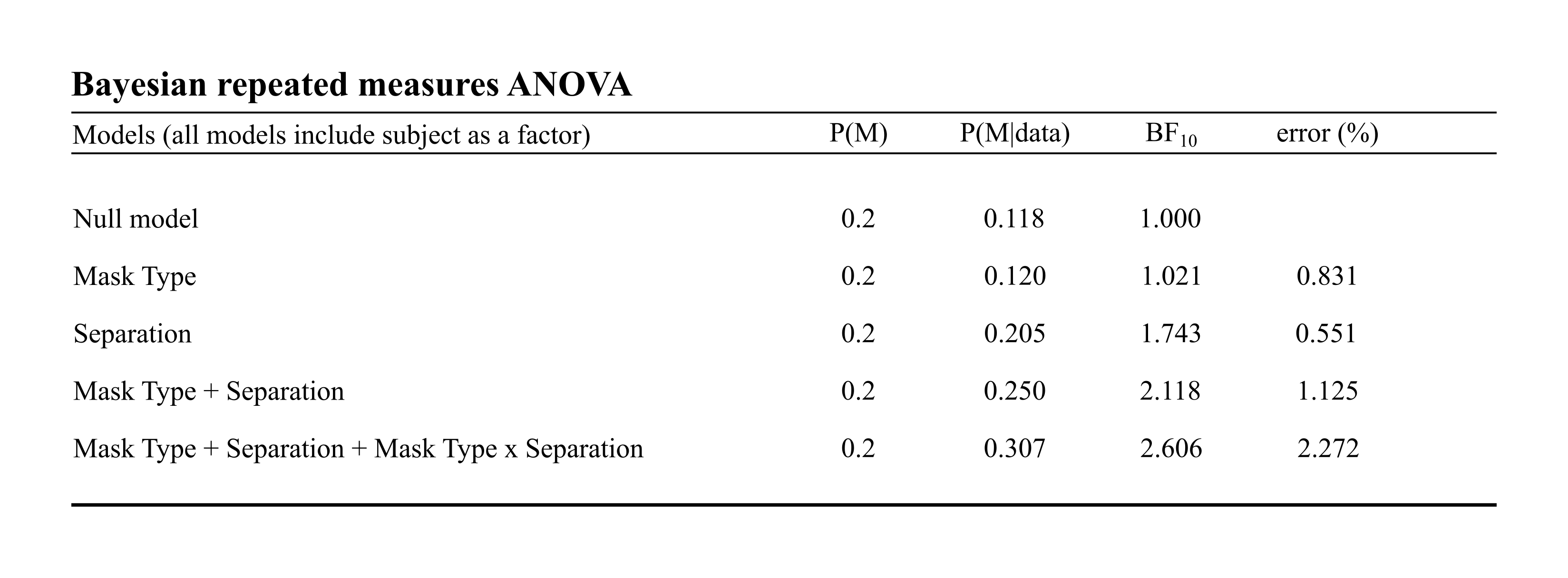

Supplement: Figure S2 — The first column indicates the model being tested. P(M) indicate prior odds; P(M|data) indicate posterior odds, and BF10 indicates the Bayes factor of the model relative to the null. Due to the transitivity of Bayes factors, models can be directly compared by taking a ratio of their Bayes factors. For example, the interaction model (BF10 = 2.606) outperforms the two factor model (BF10 = 2.118) by a factor of 2.606/2.118 = 1.23. [file peerj-04-2068-s008.png]

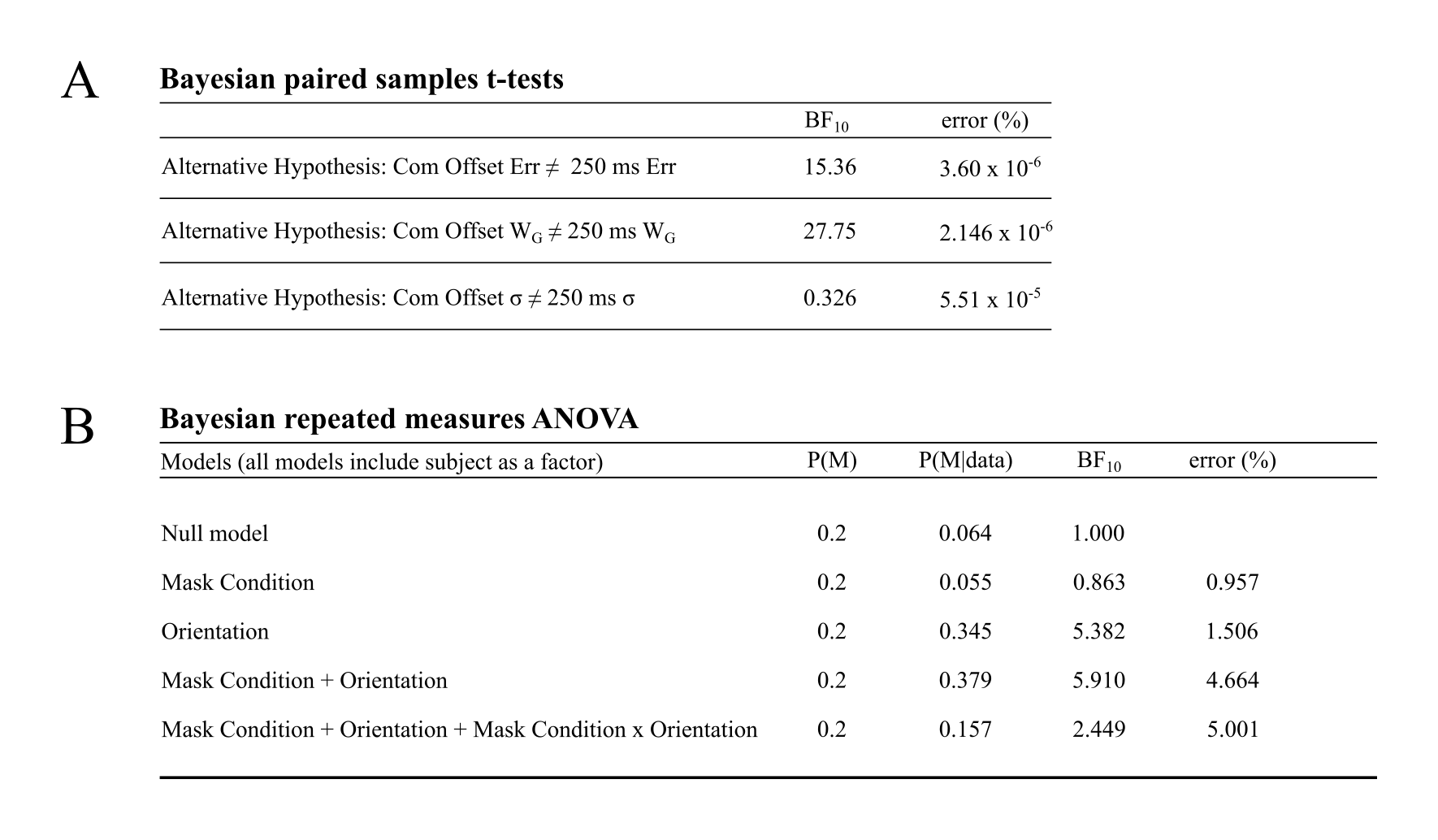

Supplement: Figure S3 — A: Three Bayeseian paired samples t-tests are shown here. The first is the overall errors between the common offset baseline and the 250 ms mask conditions. Next is the weights of the Gaussian components of the mixture model analysis between these two conditions. The third is the standard deviation of these Gaussian components. Note the Bayes factor here is with respect to the alternative hypothesis relative to the null hypothesis. The reciprocal of this value (BF01) represents the likelihood of our data given the null hypothesis relative the likelihood of our data given the alternative hypothesis. B: Results of the Bayesian repeated measures ANOVA on the effects of target orientation and masking condition. Note that the interaction model has a smaller Bayes factor than that of the two factor model. [file peerj-04-2068-s009.png]
