# Supplementary material for: A closer look at four-dot masking of a foveated target
Source: PeerJ. 2016 Jun 2;4:e2068. doi: 10.7717/peerj.2068 (PMC4893326; doi:10.7717/peerj.2068)
Supplement: Supplemental Information 2 [file peerj-04-2068-s003.jasp › index.html]

JASP 


# Results

## Bayesian Repeated Measures ANOVA

| Model Comparison - dependent | | | | | | | | | | | |
| --- | --- | --- | --- | --- | --- | --- | --- | --- | --- | --- | --- |
| Models | | P(M) | | P(M|data) | | BF M | | BF 10 | | % error | |
| Null model (incl. subject) |  | 0.200 |  | 0.118 |  | 0.534 |  | 1.000 |  |  |  |
| Mask Type |  | 0.200 |  | 0.120 |  | 0.547 |  | 1.021 |  | 0.831 |  |
| Separation |  | 0.200 |  | 0.205 |  | 1.034 |  | 1.743 |  | 0.551 |  |
| Mask Type + Separation |  | 0.200 |  | 0.250 |  | 1.330 |  | 2.118 |  | 1.125 |  |
| Mask Type + Separation + Mask Type  ✻  Separation |  | 0.200 |  | 0.307 |  | 1.772 |  | 2.606 |  | 2.272 |  |
|  | | | | | | | | | | | |
|  |  |  |  |  |  |  |  |  |  |  |  |
| --- | --- | --- | --- | --- | --- | --- | --- | --- | --- | --- | --- |
| *Note.*  All models include subject. | | | | | | | | | | | |
