# Supplementary material for: A closer look at four-dot masking of a foveated target
Source: PeerJ. 2016 Jun 2;4:e2068. doi: 10.7717/peerj.2068 (PMC4893326; doi:10.7717/peerj.2068)
Supplement: Supplemental Information 3 [file peerj-04-2068-s004.jasp › index.html]

JASP 


# Results

## Bayesian T-Test

| Bayesian Paired Samples T-Test | | | | | | | | | |
| --- | --- | --- | --- | --- | --- | --- | --- | --- | --- |
|  | |  | |  | | BF₁₀ | | error % | |
| Com Off Overall Err |  | - |  | 250 Overall Err |  | 15.36 |  | 3.602e -6 |  |
|  | | | | | | | | | |

### Inferential Plots

#### Com Off Overall Err - 250 Overall Err

##### Prior and Posterior

##### Bayes Factor Robustness Check

## Bayesian Repeated Measures ANOVA

| Model Comparison - dependent | | | | | | | | | | | |
| --- | --- | --- | --- | --- | --- | --- | --- | --- | --- | --- | --- |
| Models | | P(M) | | P(M|data) | | BF M | | BF 10 | | % error | |
| Null model (incl. subject) |  | 0.200 |  | 0.064 |  | 0.274 |  | 1.000 |  |  |  |
| Mask Condition |  | 0.200 |  | 0.055 |  | 0.234 |  | 0.863 |  | 0.957 |  |
| Orientation |  | 0.200 |  | 0.345 |  | 2.106 |  | 5.382 |  | 1.506 |  |
| Mask Condition + Orientation |  | 0.200 |  | 0.379 |  | 2.439 |  | 5.910 |  | 4.664 |  |
| Mask Condition + Orientation + Mask Condition  ✻  Orientation |  | 0.200 |  | 0.157 |  | 0.745 |  | 2.449 |  | 5.001 |  |
|  | | | | | | | | | | | |
|  |  |  |  |  |  |  |  |  |  |  |  |
| --- | --- | --- | --- | --- | --- | --- | --- | --- | --- | --- | --- |
| *Note.*  All models include subject. | | | | | | | | | | | |
