# Supplementary material for: A closer look at four-dot masking of a foveated target
Source: PeerJ. 2016 Jun 2;4:e2068. doi: 10.7717/peerj.2068 (PMC4893326; doi:10.7717/peerj.2068)
Supplement: Appendix S1 [file peerj-04-2068-s006.doc]

**Appendix**

Our study used a relatively small number of observers for each experiment. In addition to this, our finding in Experiment 3, which showed no evidence for a change in response precision with a 250 ms mask, does not allow us to meaningfully quantify the evidence that there was no change in precision. To address this, we conducted a Bayesian analysis of our data, using the free software JASP, version 0.7.5.5 (Love et al., 2015; Morey & Rouder, 2015). This software calculates the Bayes factors on distributions of effect size to assess the relative probability of observed data, between two competing statistical hypotheses (for an introduction to Bayes factors, see Jarosz & Wiley, 2014). We have included the JASP analysis files with this submission.

**Experiment 1**

To assess the evidence for and against a masking effect at a mask duration of 250 ms, we conducted a Bayesian paired samples t-test (Rouder et al., 2009) between the common offset and 250 ms conditions, with the default Cauchy prior width of 0.707 (centered on an effect size of 0). The results, shown below in **Figure A1**, show a moderate preference for the alternative hypothesis over the null hypothesis by a Bayes factor of 8.62. This supports the idea that the four dot mask had an impact upon performance.

**Experiment 2**

We ran a Bayesian repeated measures ANOVA (Rouder, et al., 2012), with mask type as the first factor: two levels (4DM, annulus); second factor = target-mask separation: four levels, excluding baseline), using the default set of priors (which assumes all five models are equally probable). The results are shown below in Figure A2. While the interaction model outperformed all the other models, relative to the null, it only outperformed the Mask Type + Separation model by a factor of 1.23, which is weak evidence in favour of this interaction. While this analysis is qualitatively consistent with our earlier analysis, it does not provide strong support for the interaction.

**Experiment 3**

We provide here three separate analyses. First, we compared the overall errors in the common offset vs. 250 ms conditions, using a Bayesian paired samples t-test. Second, we ran a Bayesian repeated measures ANOVA to test for the interaction between target orientation (oblique, cardinal) and mask condition (common offset, 250 ms). Finally, we compared the parameters of our mixture model (weight of Gaussian, and Sigma) between the common offset and 250 ms conditions using Bayesian paired samples t-tests. The same prior defaults were used as in the Bayesian analyses for the first two experiments. The results, shown below in Figure A3, are consistent with our earlier analyses. There was strong evidence for masking, with a Bayes factor of 15.36 favouring the alternative hypothesis (overall errors in common offset and 250 ms conditions are different) over the null. In the repeated measures ANOVA, the target orientation + mask condition model was favoured over the interaction model by a factor of 2.41, which is weak evidence against an interaction between target orientation and mask condition. This lends support, albeit weak, to the idea that masking here is independent of the relationship between target orientation and dot location. Finally, the paired samples t-tests showed very strong evidence that the weight of the Gaussian component was different between the two masking conditions (Bayes factor = 27.75 favouring alternative over null), and moderate evidence that the standard deviation of this Gaussian component did not change (Bayes factor = 3.07, favouring the null over the alternative). This is consistent with our earlier interpretation, and provides direct evidence that the precision of observer responses did not change between masking conditions.

**References**

Jarosz AF, Wiley J. 2014. What are the odds? A practical guide to computing and reporting Bayes Factors. *The Journal of Problem Solving* *7*(1): 2-9. DOI: http://dx.doi.org/10.7771/1932-6246.1167

Love J, Selker R, Marsman M, Jamil T, Dropmann D, Verhagen A J, Ly A, Gronau QF, Smira M, Epskamp, S, Matzke D, Wild A, Knight P, Rouder JN, Morey RD, Wagenmakers EJ. 2015. JASP (Version 0.7.5). Available at https://jasp-stats.org/user-guide/.

Morey RD, Rouder JN. 2015. BayesFactor (Version 0.9.11-3). Available at https://cran.r-project.org/web/packages/BayesFactor/index.html.

Rouder JN, Speckman, PL, Sun D, Morey RD, Iverson G. 2009. Bayesian t tests for accepting and rejecting the null hypothesis. *Psychonomic Bulletin & Review* *16*(2): 225-237.

Rouder, JN, Morey RD, Speckman PL, Province JM. 2012. Default Bayes factors for ANOVA designs. *Journal of Mathematical Psychology* *56*(5): 356-374.
